# The influence of platelet-derived products on angiogenesis and tissue repair: a concise update

**DOI:** 10.3389/fphys.2015.00290

**Published:** 2015-10-20

**Authors:** Constanza E. Martínez, Patricio C. Smith, Verónica A. Palma Alvarado

**Affiliations:** ^1^Dentistry Academic Unit, Laboratory of Periodontal Biology and Regeneration, Faculty of Medicine, Pontificia Universidad Católica de ChileSantiago, Chile; ^2^Laboratory of Stem Cells and Development, Faculty of Science, FONDAP Center for Genome Regulation, University of ChileSantiago, Chile

**Keywords:** platelet poor plasma, platelet rich plasma, angiogenesis, tissue engineering, growth factors

## Abstract

Platelet degranulation allows the release of a large amount of soluble mediators, is an essential step for wound healing initiation, and stimulates clotting, and angiogenesis. The latter process is one of the most critical biological events observed during tissue repair, increasing the growth of blood vessels in the maturing wound. Angiogenesis requires the action of a variety of growth factors that act in an appropriate physiological ratio to assure functional blood vessel restoration. Platelets release main regulators of angiogenesis: Vascular Endothelial Growth Factors (VEGFs), basic fibroblast growth factor (FGF-2), and Platelet derived growth factors (PDGFs), among others. In order to stimulate tissue repair, platelet derived fractions have been used as an autologous source of growth factors and biomolecules, namely Platelet Rich Plasma (PRP), Platelet Poor Plasma (PPP), and Platelet Rich Fibrin (PRF). The continuous release of these growth factors has been proposed to promote angiogenesis both *in vitro* and *in vivo*. Considering the existence of clinical trials currently evaluating the efficacy of autologous PRP, the present review analyses fundamental questions regarding the putative role of platelet derived fractions as regulators of angiogenesis and evaluates the possible clinical implications of these formulations.

## Introduction

Wound healing, a natural restorative response to tissue injury, is governed by an elaborate response driven by resident and circulating cells, homing to the injury site, that release soluble mediators or signals generated from the extracellular matrix (ECM; Guo and DiPietro, 2010). In adult humans, optimal wound healing involves a cascade of complex, orderly, and predictable events that include four overlapping phases: hemostasis, inflammation, proliferation, and remodeling. The adequate timing of these phases is decisive for ultimate restoration of the vascular system (Gosain and DiPietro, 2004; Eming et al., 2007). Platelets regulate hemostasis through vascular obliteration and fibrin clot formation (Guo and DiPietro, 2010). Platelets are anucleated cell fragments that originate from megakaryocytes in the bone marrow (Speth et al., 2015). Among the three-reservoir organelles described in platelets, namely lysosomes, alpha granules, and dense granules, the biggest compartments for protein storage are alpha granules. The latter are considered key organelles with respect to platelet function. In the clot, platelets are responsible for the activation and release of important biomolecules from their alpha granules, including platelet-specific proteins, growth factors, coagulation factors, adhesion molecules, cytokines, angiogenic factors, proteoglycans, and cytokines/chemokines (Nurden, 2011). The release of cytokines, chemokines, and growth factors induces proliferation and activation of the cells that are involved in wound healing such as fibroblasts, neutrophils, monocytes, smooth muscle cells, and mesenchymal stem cells (MSC) (Thushara et al., 2015).

Biological products for wound treatment and surgical interventions have been an area of enormous growth in the last two decades, as our understanding of wound healing response has increased. In particular, the use of human plasmatic fractions has been proposed to locally deliver platelet-derived factors as an autologous source of biomolecules for tissue healing. In this review, we summarize (1) the importance of growth factors and biomolecules related to angiogenesis present in plasmatic fractions with different concentrations of platelets and (2) the clinical rationale for their use in cell therapies involved in the treatment of traumatic injuries as well as degenerative diseases.

## Contribution of platelets to angiogenesis

After the inflammatory phase has been initiated, the wound healing response requires angiogenesis as a process that modulates the activation, proliferation, and migration of endothelial cells to establish new blood vessels from pre-existing vasculature (Oklu et al., 2010). Platelets play a critical role in regulating angiogenesis. Nevertheless, their contribution to blood vessel repair in the course of wound healing is still poorly understood (Eming et al., 2007; Klement et al., 2013). Alpha granules are a reservoir of biological factors for platelet physiological and pathological angiogenic responses (Peterson et al., 2010; Table 1). The release of these crucial angiogenic factors in platelet derived fraction preparations could be useful in tissue regeneration and wound healing.

**Table 1:** **Growth factors stored in platelets alpha granules involved in angiogenesis**.

**Angiogenic factor**	**Function**	**References**
Vascular endothelial growth factors (VEGFs)	Regulates angiogenesis	Min Park et al., 2014
	Controls proliferation, morphogenesis, migration, and survival of endothelial cells.	Carmeliet and Jain, 2011
	Promotes the enlargement and branching of blood vessels.	
Platelet Derived Growth Factors (PDGF)	PDGF-B and PDGF-C isoforms are involved in vessel maturation and recruitment of endothelial progenitor cells from the bone marrow.	Raz et al., 2014
	Recruits pericytes and vascular smooth muscle cells to maintain the blood vessel wall.	Dimmeler, 2005; Herbert and Stainier, 2011
Hepatocyte Growth Factor (HGF)	Mitogen for endothelial cells and stimulates secretion of VEGF.	Matsumura et al., 2013
Basic fibroblast growth factor (bFGF)	Induces proliferation and tubule formation of endothelial progenitor cells.	Litwin et al., 2015
	Stimulates secretion of VEGF on endothelial progenitors *in vitro*.	
Connective tissue growth factor (CTGF)	Regulates vascular remodeling by controlling pericyte recruitment and inducing PDGF-B expression on endothelial cells.	Hall-Gleen et al., 2012
Angiopoietins	Maintains vessel and vascular leakiness, and induces pericyte chemotaxis	Nurden, 2011; Klement et al., 2013; Hwang et al., 2015
Stromal cell derived factor (SCGF)	Induces chemotaxis of endothelial precursors and increases formation of vascular structures.	De Falco et al., 2004
Epidermal Growth Factor (EGF)	Induces tubule formation, endothelial cell proliferation, and migration.	Klement et al., 2013

## Platelet derived fractions

The use of platelet-derived fractions in tissue repair is a developing area for clinician's and researchers. Marx et al. demonstrated a potential use of Platelet Rich Plasma (PRP) in craniofacial bone grafts in the late nineties (Marx et al., 1998), and since then, plasmatic fractions have been promoted as suitable sources of autologous growth factors. PRP may be defined as a component of plasma fraction of autologous venous blood with platelet counts in the range between 4 and 6 times above baselines considered to be of therapeutic benefit (1 million platelets/L; Chen and Liu, in press). The preparation of PRP by centrifugation was initially completed by a “two-step gradient centrifugation method.” A strong first spin was used first in order to separate the erythrocytes from the clotting factors, platelets, and leukocytes. Then, the plasma was subjected to a second centrifugation step, to harvest the PRP fraction from the platelets and leukocytes. Finally, platelets in PRP were activated to release the biomolecules, using thrombin or calcium chloride. Other authors have proposed alternative methods to liberate growth factors from platelets, such as lysing the platelets by freezing them or using sonication or ultrasound (Weed et al., 2004). Once the release of biomolecules from platelets is activated, a network is formed to establish a fibrin clot that acts as scaffold for growth factors over a limited period of time (Mautner et al., 2015).

Nowadays most of the commercially accessible kits involve a one-step method to separate the plasma into three distinct layers: the erythrocytes, the buffy coat containing PRP, and the Platelet Poor Plasma (PPP) (Bausset et al., 2012; Burnouf et al., 2013). Currently, plasmatic fractions have been classified according to at least two key parameters: the presence of leukocytes and the fibrin architecture (Dohan Ehrenfest et al., 2014). Following these criteria, we can find four family fractions:

**Pure Platelet-rich Plasma (P-PRP) low or without Leukocytes:** Plasmatic preparations from anticoagulated venous blood “without leukocytes and with a low- density fibrin network.” The white blood cell count of these samples is less than the whole blood percentage (Riboh et al., 2015). After its activation with calcium chloride or thrombin, this preparation can be used as a liquid solution or as a gel (Gobbi and Vitale, 2012; Dohan Ehrenfest et al., 2014; Mautner et al., 2015).

**Platelet-rich Plasma (L-PRP) with Leukocytes:** Plasmatic fractions from anticoagulated venous blood “with leukocytes and with a low-density fibrin network.” The leukocytes content in these preparations is at least five times compared with the base line of whole blood counting (Filardo et al., 2013). Following activation, L-PRP might be used either as a liquid solution or in an activated gel form. Among the commercial systems available are Harvest Smart- PreP (Harvest Technologies, Plymouth, MA, USA), Biomet GPS III (Biomet Inc., Warsaw, IN, USA), Plateltex (Prague, Czech Republic), and Regen PRP (RegenLab, Le Mont-sur-Lausanne, Switzerland; Gobbi and Vitale, 2012; Dohan Ehrenfest et al., 2014; Mautner et al., 2015).

**Pure Platelet-rich Fibrin (P-PRF) low or without Leukocytes:** These correspond to “preparations without leukocytes and with a high-density fibrin network.” Fibrin, in combination with growth factors, has been shown to effectively support cell adhesion and proliferation. P-PRF only exists in a solid activated gel form. To date, only one product of this family is commercially available, known as Fibrinet PRFM (Platelet- Rich Fibrin Matrix, Cascade Medical, Wayne, NJ, USA; Gobbi and Vitale, 2012; Dohan Ehrenfest et al., 2014; Mautner et al., 2015).

**Platelet-rich Fibrin (L-PRF) with Leukocytes:** Also named Choukroun's PRF. In this preparation venous blood is obtained without any anticoagulant and directly centrifuged. A cascade of calcium chloride or thrombin is used, which results in the isolation of this plasmatic fraction without any biochemical modifications. These preparations, existing only in gel form, have leukocytes and a high-density fibrin network (Dohan Ehrenfest et al., 2009, 2014; Gobbi and Vitale, 2012; Mautner et al., 2015).

For a complete and updated overview of platelet-derived fractions and the requirements for their preparation for clinical use, please refer to the following other excellent reviews: De Pascale et al. (2015), Kawase (2015), and Mautner et al. (2015).

## Platelet concentrates and angiogenesis

Diverse growth factors are involved in the process of angiogenesis, of which many are secreted by platelets (Peterson et al., 2010). Given the critical role of angiogenesis in modulating wound healing and considering that platelet-derived factors are critical for vascular activation and stabilization, it is tempting to speculate whether any of the platelet-derived formulations currently used in regenerative medicine stimulate angiogenesis. Preclinical studies, using *in vitro* assays and animal models have suggested a positive influence of platelet-derived fractions on angiogenesis.

### *In vitro* studies

In order to identify the mechanisms whereby platelet-derived fractions may stimulate angiogenesis, Bertrand-Duchesne et al. (2010) evaluated the presence of angiogenic growth factors in PRP samples. They detected high levels of VEGF, PDGF-BB, EGF, and basic fibroblast growth factor (bFGF). PRP supernatants were incubated with blocking antibodies to neutralize each of these growth factors and the response to these treatments was evaluated via proliferation assays in Human Umbilical Vein Endothelial Cells (HUVEC). Notably, the use of EGF neutralizing antibodies decreased significantly the proliferation of HUVEC, while the other antibodies did not affect this response. Another mechanistic study by Mammoto et al. evaluated the role of angiopoietin-1 in driving the vascular response in mouse PRP and PRF formulations. They found that mouse platelet derived fractions containing angiopoietin-1 (Ang) were responsible for increasing proliferation, migration, and differentiation of human microvascular endothelial cells (Mammoto et al., 2013). Li et al. (2014) observed that PRP might promote vascular growth andstimulate endothelial progenitor cells to form vessel-like structures. Anitua et al. (2015) recently studied the effect of a platelet concentrate displayed within a plasma suspension that forms a fibrin matrix system on angiogenesis. This product, called PRGF (plasma rich in growth factors) (P-PRP low leukoyte content), stimulated an increase in cell proliferation and a reduction in apoptosis in primary HUVEC and skeletal myoblasts.

### Preclinical animal studies

In a recent study, Zhou et al. (2013) investigated the effect of the application of a PRP gel in open abdominal wounds performed in rats. After inducing a peritonitis lesion they performed laparotomies and animals were treated with either PPP or PRP. After 1 week of healing, the animals treated with PRP demonstrated higher blood perfusion in the original lesion as well as a more mature granulation tissue when compared to those treated with PPP. In addition, injection of the product within the injured muscle tissue of mice induced the reperfusion of blood into the lesion. Using an innovative approach to release platelet-derived growth factors in a drug-delivery system, Notodihardjo et al. (2015) used a gelatin hydrogel compound coupled to platelet rich plasma molecules to stimulate wound healing. They observed an increase in epithelialization and vascular growth when compared to other treatments, including the gelatin hydrogel system (drug delivery system) and the PRP group. In another approach to stabilize growth factors released from platelets, a fragmine-protamine micro-nanoparticle system was used to promote healing events of skin grafts showing a positive effect on wound epithelialization and angiogenesis (Takabayashi et al., 2015). Using autologous activated platelet supernatant; Kang et al. (2014) studied their effects on vasculogenesis in peripheral blood stem cells of human origin. With this approach they observed that stem cells primed with this platelet fraction stimulated vascular growth in athymic mice (Kang et al., 2014). In skin ulcers performed in porcine, Roy et al. (2011) showed that a platelet-rich fibrin matrix was able to stimulate wound healing by enhancing angiogenesis. In order to stimulate tendon regeneration, tendon healing was studied in New Zealand rabbits in which Achilles tendons were sectioned and subsequently treated with PRP or saline. PRP treated-tendons demonstrated increased angiogenesis and better collagen fiber re-alignment when compared to saline treated specimens (Lyras et al., 2009). To this end, Mammoto et al. (2013) identified that angiopoietin-1 is highly represented in PRP. Moreover, they observed that inhibition of angiopoietin-1-Tie2 signaling was able to suppress the angiogenic induction by a platelet-rich fibrin matrix *in vivo*. These studies show that in general, platelet-derived fractions may exert a potent pro- angiogenic response in different organs or anatomical locations and clearly justify further research.

## Potential aplications of platelet derived fractions: Tissue engineering

**Figure 1:**
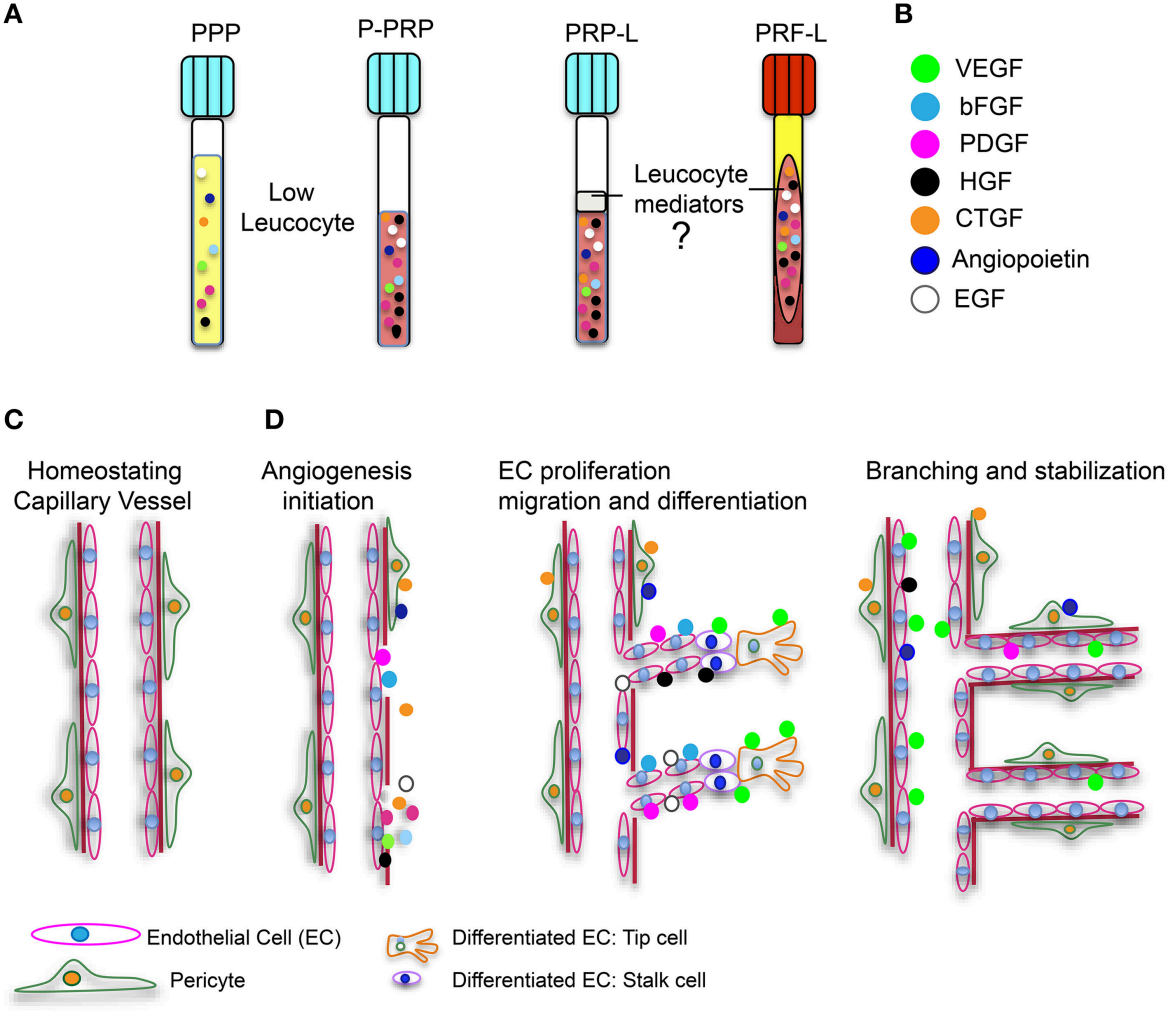
**Platelet derived fractions and involvement of angiogenic growth factors in angionesis**. **(A)** Four platelet-derived fractions are illustrated. Two with low leucocyte content (PPP and P-PRP), and two with high leucocyte quantities (L-PRP and L-PRF). **(B)** Angiogenic growth factors are represented with the indicated color code. **(C)** Illustration of a capillary blood vessel in physiological conditions. **(D)** Influence of angiogenic growth factors during angiogenesis initiation, endothelial cell (EC) proliferation, migration, and differentiation and finally, branching and stabilization of new blood vessels during a healing event.

As stated by Vishwakarma et al. (2015): “Tissue engineering and regenerative medicine is a rapidly growing multidisciplinary field involving the life, physical, and engineering sciences that seeks to repair, regenerate, or replace biological cell, tissue, and organ substitutes that have been lost due to congenital abnormalities, injury, disease, or aging.” The development of tissue-engineered products must involve a vascular support. Cellular function and viability are highly dependent on the effective diffusive exchange of nutrients through tissue. *In vivo*, cells are found within 200 μm-away from the nearest capillary network, otherwise they may suffer from ischemia and necrosis. Most of the tissue engineering scaffolds and composites are typically avascular. Therefore, it is essential that revascularization strategies stimulate regeneration of vascular networks in order to obtain a successful clinical outcome of an implanted cell-construct (Upputuri et al., 2015). The scaffolds need to support cell proliferation and differentiation to replace specific tissue loss *in vivo*. However, they must also provide a suitable substrate that allows adequate blood vessel growth to supply nutrients and oxygen to the cells located inside this engineered composite (Cenni et al., 2011). Considering the essential role of angiogenesis during the tissue engineering process, it is important to evaluate the scaffold features and properties to predict its vascularization potential and its possible interactions with endothelial and stem cells (Cenni et al., 2011). As already mentioned, plasmatic fractions constitute a source of main angiogenic growth factors, such as PDGF, VEGF, FGF-2, and EGF, as well as other proteins involved in the angiogenic process. Platelet angiogenic potential also resides in the presence of cytokines, integrins, hepatocyte growth factor, interleukin-8 (IL-8), IL-3, αvβ3− integrin 212, and matrix metalloproteinases (MMPs), which degrade ECM facilitating endothelial cell migration (Cenni et al., 2011; Klement et al., 2013). It is also important to note that the use of platelet-derived fractions has a considerable advantage, offering multiple pro-angiogenic factors compared to the application of high doses of recombinant growth factors to the wound site. Even the use of PPP could be a good alternative to the use of autologous growth factors. Recently, our research team evidenced the presence of angiogenic factors and biomolecules related to bone differentiation on PPP (Martínez et al., 2015). These results, combined with preclinical data provided by other research teams, suggest that (1) this PPP fraction could potentially be considered a good alternative to the use of autologous growth factors and proteins in combination with tissue engineering scaffolds and (2) that it might present an opportunity to increase the effectiveness of treatments in clinical applications (Yilmaz et al., 2011; Hateyama et al., 2014; Martínez et al., 2015). Angiogenic factors found in platelet-derived fractions can participate in cellular events involved in angiogenesis (Figure 1, Agren et al., 2013; Amable et al., 2013; Martínez et al., 2015). The angiogenic process requires the proliferation, migration, and adequate differentiation of endothelial cells (EC). ECs, categorized as (1) migrating leading “tip” cells to guide the direction of new blood vessel formation and (2) trailing “stalk” cells to establish the lumen of the new vessel, are indispensable to the branching and stabilization of new blood vessels (Carmeliet and Jain, 2011, Figure 1). However, it is still unknown whether or not “leukocyte mediators” have an effect on angiogenesis in the platelet-derived fractions. Overall, the use of platelet-derived fractions is promising in the process of angiogenesis during wound healing and regeneration. However, there is no consensus regarding the protocols utilized for their extraction, their effect on target cells, the concentration of the growth factors, and the effect of inflammatory mediators. Consensus needs to be reached in order to better exploit the clinical potential of platelet-derived fractions.

### Platelet derived fractions and mesenchymal stem cells (MSC)

Many preclinical and clinical studies have demonstrated the benefits of using MSC to promote tissue repair; MSC-based therapies are gaining ground in wound management. It has been shown that MSCs might repair damaged endothelium by secreting trophic factors that allow the recruitment of endogenous stem cells, which helps facilitate angiogenesis (Bronckaers et al., 2014). MSCs' activity depends on the instructive microenvironment or niche. To date, the positive influences of plasmatic fractions have been reported mainly in relation to PRP, proliferation, stemness, and preservation of the MSC immune-modulatory properties (Chieregato et al., 2011; Copland et al., 2013; for recent systematic review refer to Rubio-Azpeitia and Andia, 2014).

Additionally, translational medicine using MSC therapies requires protocols that can rule out the possibility of contamination or immunological reactions toward xenogeneic compounds (i.e., animal serum) used in traditional cell culture protocols (Lange et al., 2007; Goedecke et al., 2011). The use of fetal bovine serum in the maintenance of MSCs is undesirable because of viral/prion disease transmission risks that can initiate xenogeneic immune responses (Doucet et al., 2005; Even et al., 2006). Studies concerning bone marrow and periodontal ligament MSCs have shown that supplementing medium with autologous serum or platelet-derived fractions, instead of animal serum, is a good source of growth factors due to the fact that they provide sufficient *ex vivo* expansion, decrease the time required to reach confluence, increase the size of colony forming units, and maintain their osteogenic, chondrogenic, and adipogenic differentiation capability (Tonti and Mannello, 2008; Ben Azouna et al., 2012; Martínez et al., 2015). Advancement in stem cell research will help to reveal the intimate mechanisms and interactions between stem cells and platelet-derived growth factors in wounds.

## Final remarks

From a therapeutic viewpoint, platelet concentrate seems to be quite promising. However, there is no consensus regarding their use. The application of platelet-derived fractions still demands standardization of its preparation, a more detailed characterization of their biomolecule composition and angiogenesis potential, as well as well-designed and controlled clinical trials. Several important questions regarding the timing of treatment and the actual impact of platelet fractions on restoring angiogenic activity remain to answer.

## Funding

This work was supported by the National Fund for Scientific and Technological Development of The Chilean Government (FONDECYT) 11121294 (CM), (FONDECYT) 1130618 (PS), (FONDECYT) 1140697 (VP), and Fondo de Fomento al Desarrollo Científico y Tecnológico (FONDEF) D09E1047 (VP).

### Conflict of interest statement

The authors declare that the research was conducted in the absence of any commercial or financial relationships that could be construed as a potential conflict of interest.
